# Availability of benzathine penicillin G for syphilis treatment in Shandong Province, Eastern China

**DOI:** 10.1186/s12913-019-4006-4

**Published:** 2019-03-22

**Authors:** Xinlong Chen, Guigang Li, Yanling Gan, Tongsheng Chu, Dianchang Liu

**Affiliations:** 1 Department of STI and Leprosy Prevention and Control, Shandong Provincial Institute of Dermatology and Venereology, Shandong Academy of Medical Sciences, 27397, Jingshi Lu, Jinan, 250022 Shandong China; 2 School of Medicine and Life Sciences, University of Jinan-Shandong Academy of Medical Sciences, 6866, Jingshi Eastern Lu, Jinan, Shandong China; 3 Zhaoyuan Center for Disease Control and Prevention, 125-1, Yingbing Lu, Yantai, Shandong China

**Keywords:** Benzathine penicillin G, Syphilis, Availability, China

## Abstract

**Background:**

The shortage of benzathine penicillin G (BPG) worldwide presents a major challenge in the treatment of syphilis. Its availability for syphilis treatment has not been adequately evaluated in China.

**Methods:**

Two surveys were conducted among hospitals providing sexually transmitted infection clinical services in Shandong Province in 2012 and 2018. Data on the basic information and BPG availability of the surveyed hospitals and related factors were collected and analyzed using SPSS 17.0.

**Results:**

A total of 433 and 515 hospitals were surveyed in 2012 and 2018, respectively. A significant difference in BPG availability was observed among different levels and types of hospitals both in 2012 (X^2^ = 9.747, *p* = 0.008; X^2^ = 37.167, *p* = 0.000) and 2018 (X^2^ = 11.775, *p* = 0.003; X^2^ = 28.331, p = 0.000). The BPG availability among surveyed hospitals increased from 45.0% in 2012 to 56.4% in 2018 (X^2^ = 11.131, *p* = 0.001). The BPG availability was higher in 2018 than in 2012 among county-level hospitals (52.0% vs. 40.8%, X^2^ = 7.783, *p* = 0.005), general western medicine hospitals (62.1% vs. 50.0%, X^2^ = 6.742, *p* = 0.009), maternal and child health hospitals (57.1% vs. 26.9%, X^2^ = 13.906, *p* = 0.000), and public hospitals (56.8% vs. 45.0%, X^2^ = 11.361, *p* = 0.001). However, the county-level availability of BPG (at least one hospital has BPG in a county-level unit) has not improved between 2012 and 2018 (65.93% vs. 70.34%; X^2^ = 0.563, *p* = 0.453). The absences of clinical needs, restriction of clinical antibacterial drugs, and lack of qualifications for providing syphilis treatment were the major reasons for the low BPG availability of hospitals.

**Conclusions:**

BPG availability for syphilis treatment in Shandong Province remains low and presents disparities among different levels and types of hospitals, although it has been improved in recent years. The low availability of BPG for syphilis treatment in China is related to its clinical use by doctors rather than the market supply. Health care reforms should further improve the availability and accessibility of health services.

**Electronic supplementary material:**

The online version of this article (10.1186/s12913-019-4006-4) contains supplementary material, which is available to authorized users.

## Background

Syphilis is a chronic, multistage disease caused by *Treponema pallidum* (TP), with a global burden of 17.7 million cases and 5.6 million new infections per year [1]. One-third of untreated syphilis cases progress to late stage, resulting in profound morbidity and even death [2]. Untreated maternal syphilis can lead to serious complications, with an estimated 930,000 maternal syphilis infections that caused 350,000 adverse pregnancy outcomes in 2012 worldwide [3]. In addition, syphilis can increase the risk of acquiring HIV infection [4].

Syphilis is a unique sexually transmitted infection (STI) that remains curable with a single dose of long-acting benzathine penicillin G (BPG) without documented risk of resistance [5]. Although doxycycline, azithromycin, and ceftriaxone have the advantages of obtaining an equivalent efficacy in the treatment of early syphilis as penicillin, treating other coexisting STIs and penicillin-allergic patients, these regimens require multiple daily doses and are more cumbersome and expensive than a single dose of BPG [6–8]. Furthermore, tetracyclines are contraindicated during pregnancy [9]. Azithromycin is not recommended for the treatment of syphilis-infected fetus because it does not cross the placenta [10]. The emergence of azithromycin resistance mutations in TP has resulted in treatment failure and limited its usefulness in many regions [11]. Ceftriaxone can cross the placental barrier, but the optimal dose and duration of therapy for pregnant women are unknown and may increase the risk of kernicterus in newborns [12, 13]. No other antibiotics have superiority over penicillin for the treatment of syphilis [6]. Despite the disparity of type, dose, and duration of treatment among the guidelines across different countries around the world, penicillin is still an essential antibiotic for the treatment and prevention of syphilis [2].

However, the shortage of BPG worldwide presents a major challenge in the treatment of syphilis [14]. The World Health Organization noted that more than 3 million treatments per year globally are needed for syphilis and rheumatic heart disease [15]. Approximately 5.6 million doses of 2.4 million units of BPG are needed annually to treat all syphilis cases globally [1, 16]. Among 95 surveyed countries and territories, 39 reported a shortage of the medicine [17]. BPG shortage has affected the treatment and prevention of syphilis globally and was recognized by the 69th World Health Assembly in 2016 as an essential medicine that has been in short supply for several years [18].

China is the most populous country in the world with high syphilis disease burden. A total of 475,860 new cases of syphilis were reported in China in 2017 [19]. Accordingly, about 1–1.5 million doses of the 2.4 million units of BPG are needed annually for their treatment (2–3 doses of BPG for each case according to Chinese guideline), which may even be approximately 5.2 times more [20]. Approximately 30,882 women diagnosed syphilis during pregnancy and 30,882 live born infants needed treatment in 2012 in China [17]. China is not among the 39 countries with a shortage of BPG. However, the demand for BPG has not been consistently and adequately quantified in China. In 2013, 55.4% of the 15,884 reported pregnant women with syphilis infection in China did not receive treatment or initiated the treatment after 37 gestational weeks, and 14.0% suffered from serious adverse pregnancy outcomes [21]. What has led to the inadequate treatment of syphilis patients in Shandong? How is the availability of BPG for syphilis treatment in Shandong? We conducted a survey in 2012 and 2018 in Shandong Province to evaluate the availability of BPG for syphilis treatment and provide a basis for policy making.

## Study setting and methods

### Study site

The study was conducted in Shandong Province, China. Shandong, located on the eastern coast of China, is subdivided into 17 prefecture–level cities and 137 county–level units. By 2017, a total of 2450 hospitals had been registered in the province, including 863 public and 1587 private hospitals, where 761 are Class II and above (or county–level and above hospitals), 133 are specialized (including dermatological hospitals), 161 are maternal and child health, and 300 are Chinese medicine hospitals [22].

### Study objects

All hospitals providing STI clinical services in Shandong Province meet the criteria of inclusion. The hospitals included in the study were based on the list of those that reported syphilis cases in the past year. Hospitals under county–level, such as township hospitals and health centers, which only provide syphilis screening and referral service, were excluded from this study. Some military and enterprises hospitals were included and classified according to their scale, affiliation, and services they provide.

### Data collection

The first survey was conducted in 2012 as a part of baseline assessment for the National Program for Prevention and Control of Syphilis in China (2010–2020) [23]. Contents related to this study were covered, including basic information of the hospitals (level, type, and affiliation), availability of a department of STI services, and provision of BPG. To assess the effectiveness of the program, we conducted the second survey in 2018. In addition to the content of the first survey, related factors to the unavailability of BPG were explored. A questionnaire was used in each survey in data collection. Health staff from the Center for Diseases Control and Prevention at county–level visited each hospital in their respective jurisdictions and filled in the questionnaire (Additional file 1).

### Statistical analysis

Data collected were entered into Microsoft Excel for Windows (2012) and analyzed with SPSS 17.0 (IBM, Armonk, NY). The availability of BPG among hospitals according to levels, types, and affiliation was compared. The availability of BPG was considered when BPG was available in a hospital during the survey. Descriptive analysis and Chi–square were used for statistical analysis.

## Results

In the 2012 survey, 429 hospitals responded among 471 in 17 prefecture–level cities including 135 county–level units (counties or districts or county–level cities) that reported syphilis cases in the past year, except for 22 township hospitals or health centers. Accordingly, 433 hospitals among 515 were surveyed in 2018 in 15 prefecture–level cities including 118 county–level units, except for 125 township hospitals or health centers.

### BPG availability among hospitals at different levels in 2012 and 2018

A significant difference in BPG availability was observed among hospitals at different levels both in 2012 (X^2^ = 9.747, *p* = 0.008) and 2018 (X^2^ = 11.775, *p* = 0.003). The availability of BPG was significantly higher among provincial hospitals than among county–level hospitals both in 2012 (X^2^ = 5.613, *p* = 0.018) and 2018 (X^2^ = 9.744, *p* = 0.002). The availability of BPG was significantly higher among provincial hospitals than among municipal hospitals in 2018 (X^2^ = 4.887, *p* = 0.028) (Tables 1 and 2).

**Table 1:** BPG availability among hospitals in Shandong Province in 2012

Variables	Total (*N* = 429)	BPG		
		Yes	No	X^2^, *p* value
Level				9.747, 0.008
Provincial hospitals (n, %)	19 (4.4)	13 (68.4)	6 (31.6)	
Municipal hospitals (n, %)	96 (22.4)	52 (54.2)	44 (45.8)	
County–level hospitals (n, %)	314 (73.2)	128 (40.8)	186 (59.2)	
Types				37.167, 0.000
Specialized dermatological hospitals (n, %)	37 (8.6)	30 (81.1)	7 (18.9)	
General western medicine hospitals (n, %)	224 (52.2)	112 (50.0)	112 (50.0)	
Maternal and child health hospitals (n, %)	78 (18.2)	21 (26.9)	57 (73.1)	
Chinese medicine hospitals (n, %)	57 (13.3)	20 (35.1)	37 (64.9)	
Other specialized hospitals (n, %)	33 (7.7)	10 (30.3)	23 (69.7)	
Affiliation				0.002, 0.567
Public hospitals (n, %)	407 (94.9)	183 (45.0)	224 (55.0)	
Private hospitals (n, %)	22 (5.1)	10 (45.5)	12 (54.5)	
Dermatology department				64.920, 0.000
Yes (*n*, %)	197 (45.9)	130 (66.0)	67 (34.0)	
No (*n*, %)	232 (54.1)	63 (27.2)	169 (72.8)

**Table 2:** BPG availability among hospitals in Shandong Province in 2018

Variables	Total (*N* = 433)	BPG		
		Yes	No	X^2^, *p* value
Level				11.775, 0.003
Provincial hospitals (n, %)	22 (5.1)	19 (86.4)	3 (13.6)	
Municipal hospitals (n, %)	113 (26.1)	70 (61.9)	43 (38.1)	
County-level hospitals (n, %)	298 (68.8)	155 (52.0)	143 (48.0)	
Types				28.331, 0.000
Specialized dermatological hospitals (n, %)	22 (5.1)	19 (86.4)	3 (13.6)	
General western medicine hospitals (n, %)	232 (53.6)	144 (62.1)	88 (37.9)	
Maternal and child health hospitals (n, %)	70 (16.2)	40 (57.1)	30 (42.9)	
Chinese medicine hospitals (n, %)	80 (18.5)	33 (41.3)	47 (58.7)	
Other specialized hospitals (n, %)	29 (6.7)	8 (27.6)	21 (72.4)	
Affiliation				0.491, 0.306
Public hospitals (n, %)	405 (93.5)	230 (56.8)	175 (43.2)	
Private hospitals (n, %)	28 (6.5)	14 (50.0)	14 (50.0)	

### BPG availability among different types of hospitals in 2012 and 2018

A significant difference in BPG availability was observed among different types of hospitals both in 2012 (X^2^ = 37.167, *p* = 0.000) and 2018 (X^2^ = 28.331, *p* = 0.000). The availability of BPG among specialized dermatological hospitals was significantly higher than that among general western medicine hospitals (X^2^ = 12.367, *p* = 0.000; X^2^ = 5.159, *p* = 0.034), maternal and child health hospitals (X^2^ = 29.824, *p* = 0.000; X^2^ = 6.213, *p* = 0.020), Chinese medicine hospitals (X^2^ = 19.062, *p* = 0.000; X^2^ = 14.053, p = 0.000), and other specialized hospitals (X^2^ = 18.365, *p* = 0.000; X^2^ = 17.348, p = 0.000) both in 2012 and in 2018 (Tables 1 and 2).

In the 2012 survey, the availability of BPG among general western medicine hospitals was significantly higher than that among maternal and child health (X^2^ = 12.502, *p* = 0.000), Chinese medicine hospitals (X^2^ = 4.057, *p* = 0.044), and other specialized hospitals (X^2^ = 4.475, *p* = 0.034). (Table 1) In the 2018 survey, the availability of BPG among general western medicine hospitals was significantly higher than among Chinese medicine hospitals (X^2^ = 10.504, *p* = 0.001), and other specialized hospitals (X^2^ = 12.603, *p* = 0.000) (Table 2).

### BPG availability among hospitals by affiliation in 2012 and 2018

No significant difference of BPG availability was observed between public and private hospitals both in 2012 (X^2^ = 0.002, *p* = 0.567) and 2018 (X^2^ = 0.491, *p* = 0.306) (Tables 1 and 2).

### Changes in BPG availability among hospitals from 2012 to 2018 by level, type, and affiliation

The BPG availability among surveyed hospitals in 2018 was higher than that in 2012 (56.4% vs. 45.0%, X^2^ = 11.131, *p* = 0.001). The BPG availability was higher in 2018 than in 2012 among county-level hospitals (52.0% vs. 40.8%, X^2^ = 7.783, *p* = 0.005), general western medicine hospitals (62.1% vs. 50.0%, X^2^ = 6.742, *p* = 0.009), maternal and child health hospitals (57.1% vs. 26.9%, X^2^ = 13.906, *p* = 0.000), and public hospitals (56.8% vs. 45.0%, X^2^ = 11.361, *p* = 0.001) (Table 3).

**Table 3:** Comparison of BPG availability among the hospitals in Shandong Province in 2012 and 2018

Variables	BPG		
	2012	2018	X^2^, *p* value
Total	193 (45.0)	244 (56.4)	11.131, 0.001
Level			
Provincial hospitals (n, %)	13 (68.4)	19 (86.4)	1.916, 0.260
Municipal hospitals (n, %)	52 (54.2)	70 (61.9)	1.293, 0.255
County-level hospitals (n, %)	128 (40.8)	155 (52.0)	7.783, 0.005
Types			
Specialized dermatological hospitals (n, %)	30 (81.1)	19 (86.4)	0.274, 0.729
General western medicine hospitals (n, %)	112 (50.0)	144 (62.1)	6.742, 0.009
Maternal and child health hospitals (n, %)	21 (26.9)	40 (57.1)	13.906, 0.000
Chinese medicine hospitals (n, %)	20 (35.1)	33 (41.3)	0.533, 0.465
Other specialized hospitals (n, %)	10 (30.3)	8 (27.6)	0.055, 0.814
Affiliation			
Public hospitals (n, %)	183 (45.0)	230 (56.8)	11.361, 0.001
Private hospitals (n, %)	10 (45.5)	14 (50.0)	0.102, 0.749

### Availability of BPG among county–level units in 2012 and 2018

If BPG was available in at least one hospital in a county-level unit, BPG was considered available in the county-level unit. BPG was available in 65.93% (89/ 135) county-level units in 2012 and 70.34% (83/118) in 2018. No significant difference in BPG availability at county-level was observed between 2012 and 2018 (X^2^ = 0.563, *p* = 0.453).

### Related factors to BPG availability for syphilis treatment in 2018

In the 2018 survey, we explored the reasons why BPG was unavailable in 189 (43.6%) hospitals. Three major answers were provided by 181 hospitals. The primary reason was the absence of clinical needs (72/181, 38.5%). Second, the clinical use of antibacterial drugs was restricted (37/181, 30.5%). Finally, qualifications lack for providing syphilis treatment (32/181, 27.8%), that is, a qualified department of dermatology & STI and respective doctors are lacking. Among 429 hospitals in the 2012 survey, 197 (45.92%) had a department of dermatology & STI. The availability of BPG among hospitals that have a department of dermatology & STI was significantly higher than those without (66.0% vs. 27.2%, X^2^ = 64.920, *p* = 0.000) (Table 1).

## Discussion

The Chinese government issued the National Program for Prevention and Control of Syphilis in China (2010–2020) in 2010 [23]. The targets of the program are achievable by 2020 given that BPG can cure most syphilis cases. The availability of BPG for syphilis treatment in Shandong Province has improved in the past 5 years. However, BPG was only available in 56.4% of the hospitals and still had a low availability of 70.3% at the county level in 2018, which was still far from the targets of the Chinese government.

We found a significant difference in BPG availability among different types of hospitals. Specialized dermatological hospitals still play an important role in STI and leprosy prevention and control in China. Maternal and child health hospitals should play a vital role in the prevention and treatment of maternal and congenital syphilis. Although significant change has been observed in the past 5 years, they are still vulnerable in BPG availability.

The low BPG availability among maternal and child health, Chinese medicine, and other specialized hospitals was mostly due to the lack of qualifications for providing syphilis treatment or the lack of a department of dermatology & STI. We found a significant difference in BPG availability between hospitals with and without department of dermatology & STI. For general western medicine hospitals, the major reasons for the lack of BPG are the absence of clinical needs and the restriction of the clinical use of antibacterial drugs.

However, these reasons are untenable. Syphilis has been the third most prevalent disease among the second–class infectious diseases stated by the Infectious Disease Prevention Act of China and is common in general clinical practice [19, 24]. An underlying reason for the absence of clinical needs is that clinical doctors other than dermatologists lack confidence, are reluctant to manage syphilis cases, and would rather refer them to other hospitals if they have not received sufficient training. Second, penicillin has the disadvantage of “high” rate of allergy, which probably prevents doctors to use BPG, considering the great tension in patient-doctor relationships. Although the prevalence of patient–reported penicillin allergy in the general population is as high as 10, 90% of penicillin-allergic patients are able to tolerate penicillin [25]. Although some people are really allergic to penicillin, penicillin therapy may be given to pregnant women with penicillin allergy and serious infections with an acceptable level of safety by using desensitization procedures [9, 26]. Third, many alternative drugs may prevent doctors using BPG. Due to the performance assessment in Chinese hospitals, some doctors prefer second line medicines, such as cephalosporins and macrolides, over BPG, because the low-cost of BPG cannot make profits for hospitals and doctors. A consequence of avoiding the most effective first–line medicines is inducing bacterial resistance [11].

The first version of the National Essential Drugs List was issued by China’s Ministry of Health in 2009 [27]. Until 2012, BPG was excluded from the updated version [28]. Essential drugs should be prepared by all healthcare facilities and are used and sold almost exclusively. However, BPG was still unavailable in many hospitals in Shandong Province. In our 2018 survey, 30.5% of the hospitals lacking BPG refused to purchase it due to the restriction of the clinical use of antibacterial drugs. BPG used to be listed in the restricted antibacterial drugs for clinical use in China. However, the China Ministry of Health excluded it from the list in 2015 in view of its availability [29].

The barriers of BPG unavailability in China were different from those in other countries that suffer from BPG shortage due to market supply [17]. Globally, three companies that still produce the active pharmaceutical ingredient for BPG are located in China. The market supply of BPG is adequate in China. Many hospitals responded to our survey that they can restock BPG at any moment when needed. The implication of our study to other developing countries with shortage of penicillin is that many factor may related to BPG availability beside market supply.

To understand and resolve the dilemma of BPG availability for syphilis treatment in China, the healthcare system reform dated from 1990s should be introspected. Chinese public hospitals are the most important health facilities. Private parties are encouraged to provide medical service and cooperate with public sectors [30]. Hospital reforms aim to maintain the social welfare nature of public hospitals and encourage them to perform public service functions [31]. However, the market-oriented reform distorted the social welfare nature of public hospitals, keeping basic wages low for doctors and encouraging them to make money from prescriptions, which leads to perverse incentives and inequity in healthcare services [32]. The problem may be resolved with the expansion of health system reforms. In 2017, public hospital reforms expanded their focus on eliminating the drug price difference between hospital pharmacies and wholesales [33]. Furthermore, more policy oriented, legal and technical supports should be given to hospitals and clinical doctors to encourage them to restock and use BPG. The 13th Five-Year Plan for Deepening the Medical and Health System Reform states that private parties are encouraged to provide medical service and cooperate with public sectors [31]. In the future, private sectors may contribute more to the availability and accessibility of health services in China.

### Limitations of the study

First, the study was conducted in Shandong Province, one of the most economically developed provinces and the second most populous province in China. Although it covers 1/14th of the total population in China, the results of this study may be representative of the coastal economically developed areas in China. Second, this study focused on the availability rather than the accessibility and affordability of BPG. The latter are also important factors for the equity of health care delivery, and need further investigation.

## Conclusions

BPG availability for syphilis treatment in Shandong Province remains low and presents disparities among different levels and types of hospitals, although it has been improved in recent years. The low availability of BPG for syphilis treatment in China is related to its clinical use by doctors rather than the market supply. Health care reforms should further improve the availability and accessibility of health services.

## Additional file

Additional file 1: Questionnaire on availability of benzathine penicillin G in medical institutions. (DOCX 13 kb)

## Abbreviations

BPG: benzathine penicillin G
STI: sexually transmitted infection
TP: *Treponema pallidum*
