# Supplementary material for: Availability of benzathine penicillin G for syphilis treatment in Shandong Province, Eastern China
Source: BMC Health Serv Res. 2019 Mar 22;19:188. doi: 10.1186/s12913-019-4006-4 (PMC6431030; doi:10.1186/s12913-019-4006-4)
Supplement: Supplementary file 1 — Questionnaire on availability of benzathine penicillin G in medical institutions. (DOCX 13 kb) [file 12913_2019_4006_MOESM1_ESM.docx]

**Questionnaire on availability of benzathine penicillin G in medical institutions**

Code________________

Name of medical institution__________________________________

**1.Level of hospital:** ①Provincial ②Municipal ③County-level

**2.Type** ①Specialized dermatological hospitals ②General western medicine hospitals ③Maternal and child health hospitals ④[Chinese medicine hospital](javascript:;)s ⑤other

**3. Affiliation** ①Public ②Private ③other ______________________

**4. A**vailability of a department of STI services ① Yes ②No

**5. A**vailability of **benzathine penicillin G**

①Yes ②No([reason](javascript:;)) __________________________

Contact person_________________

Contact telephone Number_________________

Date of survey_________________
